# Probationers in Excess

**Published:** 1908-07-25

**Authors:** 


### Nursing Administration.

### 1.—PROBATIONERS IN EXCESS.

The art of economy in institutions is inseparably connected with the organisation of the staff. Over parsimony in numbers results inevitably in extravagance, since it promotes slovenly methods and places a bar on supervision. The poor administrator proportions the staff amiss, so that while too many hands are employed in one department, another is hampered for lack of proper attention. There can be no denying the fact that some matrons organise the nursing staff better than others. They study the day's routine in every branch of the work right through the hospital, and succeed in getting smooth results with the smallest expenditure of labour. Their "genius for organisation," closely examined, turns into the old definition of genius, "a capacity for taking pains." For those who are dissatisfied with their own results there can be no better course than to study the methods of others. The statistics published this year in "Burdett's Hospital Annual" are well worth the earnest consideration of all on whom the task of organisation depends. Comparison is always difficult because circumstances differ, but it is often a good thing to lay aside for the moment numerous and minute differences in hospital organisation, and study the particulars which all have in common. The annexed comparative tables are based on the principle of deducting from the staff the heads of departments and nurses exclusively engaged outside the wards, in out-patient duty, in electric light, massage, etc., in the home department, and household duties. The number of occupied beds is then divided among the remaining day and night staff, which includes all nurses detailed for special duty, as well as those on holiday, or used as reserves. The percentage of first year probationers and paying pupils has been carefully reckoned on the total nursing staff, so that it is apparent at a glance how many raw hands imperfectly acquainted with their duties the matron has to cope with out of every hundred nurses.

It is an interesting point in connection with these statistics to consider the normal ratio of new hands in a hospital. In institutions offering a course of four years' training it might be concluded that the percentage of first year probationers could not rise above twenty if no interruption occurred to the completion of each probationer's course. For if 20 per cent. of probationers enter each year and remain for four years, in four years' time 80 per cent. of the staff will consist of nurses under training, and but 20 per cent. can have held their positions longer than four years. And on the same principle hospitals offering a three years' course will admit as new probationers 25 per cent. of the whole staff every year, 75 per cent. of the staff being thus always in training, and the remaining 25 per cent. sisters and fully-trained nurses. An examination of the figures above will reveal, however, that these calculations are not borne out by the facts. The percentage of new probationers amounts in every case among London Hospitals, except at the Royal Free and at the Charing Cross Hospitals, to over 30 per cent. of the total nursing staff, and in seven provincial hospitals this is the case also. This high percentage of learners in the staff may result in two ways. Either interruption occurs in the training of probationers, which leads to unexpected vacancies, or the percentage of more permanent members of the staff, sisters, matrons' assistants, etc., falls below the natural average of 20 or 25 per cent.

*(To be continued.)*

| Name | Daily Average No. of Occupied Beds in 1906 | Total Nursing Staff | Average No. of Beds to each Member of Day and Night Staff | Paying Probationers | Percentage in Staff of First Year Probationers and Pupils |
|---|---|---|---|---|---|
| **Medical Schools—*London*.** | | | | | |
| London ... ... ... ... | 790 | 436 | 2.1 | 12 | 31.6 |
| St. Bartholomew's ... ... | 556 | 288 | 2.0 | * | * |
| Guy's ... ... ... ... | 520 | 267 | 2.1 | 31 | 37.8 |
| St. Thomas's ... ... ... | 475 | 189 | 2.6 | 25 | 31.7 |
| St. George's ... ... ... | .329 | 148 | 2.3 | — | 58.1 |
| Middlesex Hospital (including Cancer Wing) ... ... ... | 309 | 135 | 2.5 | 3 | 31.8 |
| St. Mary's... ... ... ... | 253 | 104 | 2.8 | 4 | 32.6 |
| University College ... ... | 242 | 99 | 2.7 | 7 | 32.3 |
| King's College ... ... ... | 188 | 86 | 2.3 | 3 | 31.3 |
| Royal Free ... ... ... | 149 | 56 | 2.8 | 1 | 16.0 |
| Charing Cross ... ... ... | 161 | 61 | 3.0 | — | 13.1 |
| ***Provincial.*** | | | | | |
| Leeds General Infirmary ... | 365 | 103 | 3.8 | — | 24.2 |
| Birmingham General Hospital | 304 | 106 | 3.2 | — | 22.6 |
| Sheffield Royal Infirmary ... | 227 | 71 | 3.4 | — | 22.5 |
| Liverpool Royal Southern ... | 168 | 51 | 3.5 | — | 17.6 |
| Oxford, Radcliffe Infirmary ... | 117 | 41 | 3.3 | 2 | 24.3 |
| Cambridge, Addenbrooke's Hospital... ... ... ... | 105 | 35 | 3.5 | — | 22.8 |
| ***Scotch.*** | | | | | |
| Edinburgh Royal Infirmary ... | 839 | 246 | 3.6 | — | 24.3 |
| Glasgow Royal Infirmary ... | 580 | 161 | 4.0 | — | 19.8 |
| Glasgow Western Infirmary ... | 469 | 170 | 2.9 | — | 17.6 |
| Dundee Royal Infirmary ... | 248 | 103 | 2.5 | — | 19.4 |
| ***Irish.*** | | | | | |
| Belfast Royal Victoria Hospital | 218 | 77 | 3.2 | — | 27.2 |
| **General Hospitals—*London*.** | | | | | |
| Seamen's Hospital ... ... | 250 | 52 | 5.4 | 2 | 26.9 |
| Great Northern Central ... | 145 | 56 | 2.9 | — | 25.0 |
| German ... ... ... ... | 118 | 21 | 6.5 | — | — |
| Poplar ... ... ... ... | 78 | 37 | 2.3 | 1 | 29.7 |
| Bolingbroke ... ... ... | 31.8 | 15 | 2.6 | — | 26.6 |
| Miller ... ... ... ... | 19.2 | 11 | 2.7 | — | 36.3 |
| ***Provincial.*** | | | | | |
| Norfolk and Norwich ... ... | 168 | 55 | 3.5 | — | 23.6 |
| Sunderland Infirmary... ... | 178 | 49 | 4.1 | — | 28.5 |
| Wolverhampton and Staffs .. | 144 | 49 | 3.4 | — | 24.4 |
| Leicester Infirmary ... ... | 176 | 66 | 2.9 | 3 | 25.7 |
| Stoke-on-Trent, N. Staffs Inf. | 176 | 50 | 3.8 | — | 20.0 |
| Exeter, Royal Devon & Exeter | 162 | 65 | 2.7 | — | 30.7 |
| Cardiff Infirmary ... ... | 160 | 52 | 3.4 | 4 | 17.3 |
| Northampton General... ... | 144 | 43 | 4.0 | — | 20.9 |
| Reading, Royal Berkshire ... | 140 | 51 | 3.0 | 2 | 39.2 |
| Royal Portsmouth Hospital ... | 123 | 37 | 3.9 | — | 18.9 |
| Plymouth... ... ... ... | 120 | 47 | 2.8 | 7 | 14.9 |
| Preston and Co. Lancs Royal Infirmary ... ... ... | 104 | 29 | 4.1 | — | 20.6 |
| Worcester General Infirmary... | 102 | 34 | 3.4 | — | 23.5 |
| Ipswich, E. Suffolk & Ipswich | 96 | 30 | 3.5 | — | 20.0 |
| York County ... ... ... | 95 | 39 | 2.8 | 4 | 30.7 |
| Chester General... ... ... | 93 | 42 | 2.4 | — | 21.4 |
| Gloucester General Infirmary | 84 | 34 | 2.9 | — | 35.2 |
| Canterbury, Gen. Kent & Cant. | 74 | 23 | 3.5 | — | 34.7 |
| Stockport Infirmary ... ... | 73 | 21 | 3.8 | — | 23.8 |
| Guildford Roy. Surrey Co. Hos. | 67 | 21 | 3.5 | — | 38.0 |
| Cheltenham General Hospital | 59 | 28 | 2.2 | — | 39.2 |
| Herefordshire General Hos. ... | 57 | 20 | 3.1 | — | 35.0 |
| ***Scotch.*** | | | | | |
| Kilmarnock Infirmary... ... | 97 | 22 | 4.8 | — | 9.0 |
| Dumfries and Gall. Royal Inf. | 77 | 25 | 3.3 | — | 24.0 |

